# Synergistic Improvement in Wheat Yield, Water and Nitrogen Use Efficiency in Wheat–Maize Rotation Systems: A Meta-Analysis of Multidimensional Agricultural Practices

**DOI:** 10.3390/plants15040617

**Published:** 2026-02-15

**Authors:** Huihui Wei, Tingting Gong, Li Zhou, Li Qin

**Affiliations:** 1 Institute for Interdisciplinary Innovation Research, Xi’an University of Architecture and Technology, Xi’an 710055, China; 2 Institute of Agro-Environmental Protection, Ministry of Agriculture and Rural Affairs, Tianjin 300191, China

**Keywords:** agricultural practices, yield, nitrogen use efficiency, water use efficiency, fertilization

## Abstract

Agricultural practices (APs) comprehensively regulate crop growth; however, comprehensive studies evaluating the effects of APs on crop yield, water use efficiency (WUE), and nitrogen use efficiency (NUE) remain scarce, particularly regarding determining optimal APs for winter wheat in wheat–maize rotation systems. Here, this study conducted a meta-analysis based on 305 studies globally (4009 pairs of observations), focusing on five APs: irrigation, fertilization, tillage, residue utilization, and mulching. And the results indicated that APs significantly increased winter wheat yield (31.1%), NUE (14.7%), and WUE (27.6%), with fertilization showing the most pronounced effects at 43.7%, 16.9%, and 44.7%, respectively. Specifically, compared to no fertilization, combined organic and mineral fertilizer produced the highest yield increase (141.5%); among conventional fertilization, biochar addition showed the best yield increase (19.1%). Slow-controlled/-release fertilizer and inhibitor addition increased NUE by 17.7% and 26.6%, respectively, and residue utilization and mulching improved WUE (by 17.3% and 33.2%). Moreover, in cold and arid regions (mean annual temperature [MAT] < 13 °C and total annual precipitation [TAP] < 550 mm), APs showed stronger promotion of wheat yield and WUE, while in warm and humid regions, the increase in NUE was more significant (15.3–16.1%). When experiment duration was ≥5 years, APs resulted in the highest yield increase (47.9%), while NUE and WUE increased in short-term experiments. Although APs with high nitrogen application rates resulted in a greater yield increase (51.5%), fertilization significantly reduced NUE above 198 kg N ha^−1^. Structural equation modeling revealed that, among APs, climatic conditions, soil properties, and management factors, APs were the primary driver of changes in yield and WUE, while NUE was mainly regulated by management factors. Overall, these findings provided an empirical basis for optimizing agricultural practices in wheat–maize systems and offer guidance for developing site-specific policy design.

## 1. Introduction

Wheat (*Triticum aestivum* L.) is a core component of the global agricultural production, accounting for approximately 21% of the world’s food supply [1,2], with the stability and sustainability of its production directly impacting global food security [3]. With continued population growth and changes in dietary structure, global wheat demand is projected to increase by approximately 60% by 2050 compared to current levels, exacerbating supply–demand imbalances [4,5]. Meanwhile, current wheat production faces multiple challenges, including climate change, water scarcity, and limited arable land resources [6,7], so how to achieve sustained wheat yield growth through sustainable and intensive production pathways has become a core issue urgently needing to be addressed in the global agricultural sector.

Water and nitrogen are the two most critical limiting factors determining wheat yield [8,9]. Irrigated agriculture consumes approximately 70% of the world’s freshwater resources, while nitrogen fertilizer application contributes more than 60% to grain yield increases, highlighting their central role in modern agricultural production [10,11]. Currently, in many wheat-producing areas, farmland management still prioritizes high yields, leading to widespread extensive water use and excessive nitrogen fertilizer application [12], not only wasting precious water resources, but also triggering a series of severe environmental issues, such as groundwater nitrate pollution, greenhouse gas emissions, and soil acidification [13]. Therefore, enhancing water and nitrogen use efficiency in wheat production to synergistically achieve the goals of “high yield, high efficiency, and environmental protection” is an imperative for sustainable agricultural development.

To address these challenges, a series of agricultural practices (APs) aimed at improving wheat water and nitrogen efficiency, such as fertilization (slow-controlled/-release fertilizer, combined organic and mineral fertilizer, organic fertilizer, and inhibitor addition), mulching (plastic film mulching, grass mulching), residue utilization (straw mulching, straw incorporation), and tillage have been widely proposed and applied. Meanwhile, as a highly resource-efficient cropping pattern, the winter wheat–summer maize rotation system holds an extremely important position in global agricultural production, and winter wheat, as a key crop, is crucial; its water and nitrogen management not only affects the yield and resource use efficiency but also plays a vital role in the overall sustainability of the system [14]. Therefore, clarifying the response patterns of winter wheat to various agricultural practices within the system is beneficial for promoting the efficient use of resources under intensive cropping systems.

The use of field experiments conducted in specific locations and seasons has long been a fundamental method for optimizing wheat production practices [15,16]. However, extrapolating results beyond the local sites and climatic conditions of these experiments may lead to unreliable conclusions [17]. Therefore, conclusions based on individual trials make it difficult to form universally applicable guidelines. In this context, meta-analysis, as a powerful quantitative synthesis method, can systematically integrate and quantify independently published research results globally, thereby revealing universal patterns at a higher dimension and identifying key environmental and management drivers that contribute to heterogeneity among studies [18,19]. Although some studies have conducted meta-analyses on specific practices (e.g., mulching or organic fertilizer substitution) [20,21,22], a systematic, comprehensive study to compare the synergistic effects of different agricultural practices on winter wheat yield, water use efficiency, and nitrogen use efficiency in wheat–maize systems, and to clarify their optimal application conditions, remains lacking.

On this basis, this study achieved the following core objectives by constructing a comprehensive global dataset and employing meta-analysis (1) to quantitatively assess the average effects of different agricultural practices (fertilization, irrigation, mulching, residue utilization, and tillage) on winter wheat yield, water use efficiency (WUE), and nitrogen use efficiency (NUE) in the wheat–maize system; (2) to reveal how climatic factors (e.g., mean annual temperature [MAT] and total annual precipitation [TAP]), soil properties (e.g., soil organic carbon [SOC], pH, and bulk density [BD]), and field management details (e.g., nitrogen application rate [Nrate] and experimental duration) modulate the actual effects of APs; and (3) to investigate the main driving factors affecting the relative change in winter wheat yield, NUE, and WUE. Ultimately, this study aimed to provide scientific evidence and data support for optimizing the water–nitrogen co-management technology for winter wheat in wheat–maize systems in different agroecological regions, thereby contributing wisdom to advancing the sustainable development of global winter wheat production.

## 2. Methods

### 2.1. Data Collection

We searched for peer-reviewed research articles published between 2000–2025 which reported the paired yield, NUE, and WUE responses to APs on the Web of Science (https://www.webofscience.com, accessed on 30 September 2025) and the China National Knowledge Infrastructure Database (http://www.cnki.net/, accessed on 30 September 2025). The literature search adhered to the guidelines outlined by the Preferred Reporting Items for Systematic Reviews and Meta-Analyses (PRISMA) [23] (Figure S1), and the latest date was September 2025. And we used the following string: TS = (“yield” OR “NUE” OR “nitrogen use efficiency” OR “WUE” OR “water use efficiency”) AND TS = (“tilla*” OR “irrigat*” OR “fertiliz*” OR “straw” OR “residue*” OR “mulch*”) AND TS = (“wheat” OR “winter wheat”).

In addition, we established the following criteria to further screen the completeness, relevance, and scientific value of the articles: (1) the literature is based on field studies (i.e., no pot, greenhouse, review, or model studies); (2) the planting system is a wheat–maize rotation system, and the duration should be greater than or equal to one winter wheat growing season; (3) the literature must contain well-defined treatment and control experiments, and every treatment must include three or more replicates; (4) the literature needs to contain yield, NUE, or WUE and report the mean values, standard deviation, and replicate size; and (5) when the literature reports several independent experiments of AP, each experiment will be regarded as an independent data record.

**Figure 1:**
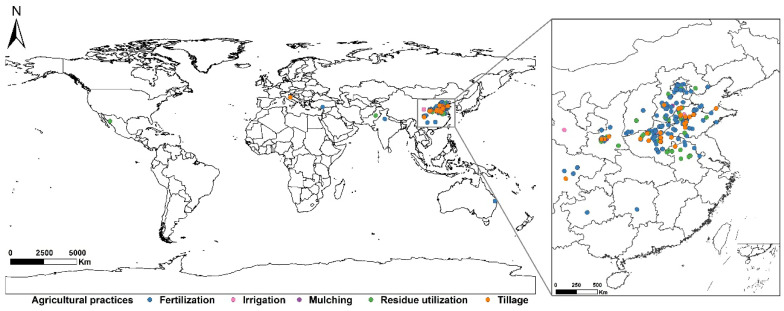
Locations of study sites included in this meta-analysis.

Finally, we screened to obtain 305 peer-reviewed research articles, including 4009 original records—3368 pairs of yield, 216 pairs of NUE, and 425 pairs of WUE (Figure 1), mainly from China (3894), India (88), Italy (10), Turkey (7), Mexico (6), and Australia (4). Based on our dataset, we classified APs into five types, i.e., tillage, irrigation, fertilization, residue utilization, and mulching. We further categorized the agriculture types into 11 subgroups, and more details are provided in Table 1. Here, we incorporated mulching into our study, covering both plastic film mulching and grass mulching. Plastic mulching, as a widely adopted agricultural technique in China, plays a crucial role in boosting crop yields, improving soil hydrothermal conditions, and suppressing weed growth, making a significant contribution to ensuring food security and stable agricultural production [24,25]. Simultaneously, this technology has accumulated extensive scientific research, forming a relatively systematic theoretical and practical base, thereby holding significant practical implications and research value [26,27,28].

### 2.2. Data Analysis

This study used meta-analysis of response ratios (*RR*) to evaluate the effect sizes of APs on winter wheat yield, NUE, and WUE. The RR was calculated as [29]

(1) $$RR=\ln\left(X_{t}-X_{c}\right)=\ln X_{t}-\ln X_{c}$$

where *X_t_* and *X_c_* are the mean value of the treatment group and control group, respectively. Variance was calculated as

(2) $$V(RR)=\frac{SD_{t}^{2}}{N_{t}X_{t}^{2}}+\frac{SD_{c}^{2}}{N_{c}X_{c}^{2}}$$

where *SD_t_* and *SD_c_* are the standard deviation, and *N_t_* and *N_c_* are the sample size of the treatment group and control group, respectively. When *SD* was not given in the study, but standard error (*SE*) was used instead, the following equation is used to convert the latter to *SD*:

(3) $$SD=SE\times \sqrt{n}$$

And for studies that did not give *SD* or *SE*, *SD* was calculated from 1/10 of the mean values [30].

The restricted maximum likelihood (REML) random effects model was carried out by the “metafor” package (version 2.4.0) to calculate the mean weighted response ratio (RR_++_) and 95% confidence intervals (CI). If the 95% CI does not overlap with zero, the response of yield, NUE, and WUE to APs can be considered significant (*p* < 0.05). To interpret effect size more easily, we further convert the response ratio into the change

(4) $$\mathrm{Relative}\;\mathrm{change}(\%)=(e^{RR_{++}}-1)\times 100$$

where *Var* representing yield, NUE, or WUE.

Here, we conducted a two-stage meta-analysis. In the first stage, we calculated the relative changes in wheat yield, NUE, and WUE under different APs. Simultaneously, based on subgroup analysis, we explored the impact of different climate and soil variables on these changes. The climate and soil variables included MAT, TAP, SOC, pH, and BD—all critical factors influencing crop growth [20,31]. Subgroup divisions for these variables referenced the work of Liu et al. (2021) [32]. In the second stage, we conducted a meta-regression analysis combining climate and soil variables to explore how the relative changes in yield, NUE, and WUE under the APs varied with soil and climate variables.

### 2.3. Statistical Analysis

The fail-safe coefficient was used to detect the presence of publication bias using the “metafor” package (version 2.4.0); detailed information can be found in Text S1, and there was no potential publication bias in our dataset (Table S1). To clarify the driving factors affecting yield, NUE, and WUE changes under agricultural practices, we constructed a multivariate index using principal component analysis (PCA) and built a structural equation model (SEM) [33]. This SEM comprehensively considered environmental, soil, and management factors, as well as agricultural practices. PCA used the “prcomp()” function, and the SEM used the “lavaan” (0.6–10) package. Information on the SEM fit index can be found in Text S2. Statistics were run using R software (version 4.2.1), and spatial maps were generated using ArcGIS (version 10.6).

## 3. Results

### 3.1. Data Overview

**Figure 2:**
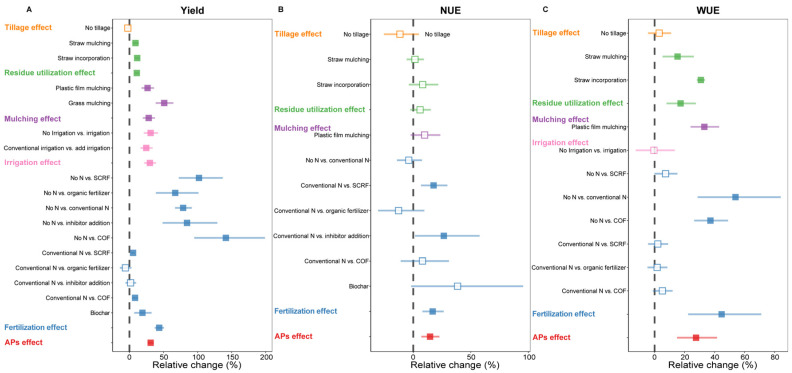
The relative change (%) in winter wheat yield (**A**), NUE (**B**), and WUE (**C**) depending on agricultural practices (APs). Yellow, green, purple, pink, and blue represent tillage, residue utilization, mulching, irrigation, and fertilization practices, respectively; red represents all agricultural practices in summary. Squares with error bars present the means ± 95% confidence intervals, and the solid symbols indicate significant response to agricultural practices; hollow symbols indicated non-significant effects. COF, combined organic and mineral fertilizer. SCRF: slow-controlled/-release fertilizer. NUE, nitrogen use efficiency. WUE, water use efficiency.

Overall, APs significantly increased winter wheat yield by 31.1% (95% CI: 26.7–35.7%, Figure 2A), and fertilization had the most pronounced effect on yield, with an average increase of 43.7%. Specifically, compared with no-fertilization, the combined organic and mineral fertilizer (COF) showed the largest increase in yield, reaching 142% (95% CI: 95.1–199%), and among conventional fertilization, biochar addition showed the most significant yield increase, with an increase of 19.1% (95% CI: 7–32.5%). Irrigation and mulching also significantly increased winter wheat yield by 30% and 28.2%, respectively.

Compared to their effects on yield, APs had a relatively limited effect on wheat NUE (14.7%, Figure 2B). A significant increase in NUE was observed only under fertilization (16.9%), with slow-controlled/-release fertilizer (SCRF) and inhibitor additions increasing NUE by 17.656% (95% CI: 6.7–29.7%) and 26.6% (95% CI: 1.6–57.7%), respectively, while other APs showed no significant effect (*p* > 0.05).

Regarding WUE, APs increased it by 27.6%, with fertilization having the greatest impact at 44.7% (Figure 2C). Compared to no fertilization, conventional nitrogen fertilizer and COF increased WUE by 53.9% and 37.2%, respectively (*p* < 0.05). Additionally, residue utilization also significantly enhanced winter wheat WUE, with straw incorporation (30.9%, 95% CI: 28.2–33.6%) outperforming straw mulching (15.3%, 95% CI: 5.3–26.2%). And plastic film mulching also increased winter wheat WUE by 33.2%.

### 3.2. Response of Yield, NUE, and WUE to Climate Factors Under APs 

**Figure 3:**
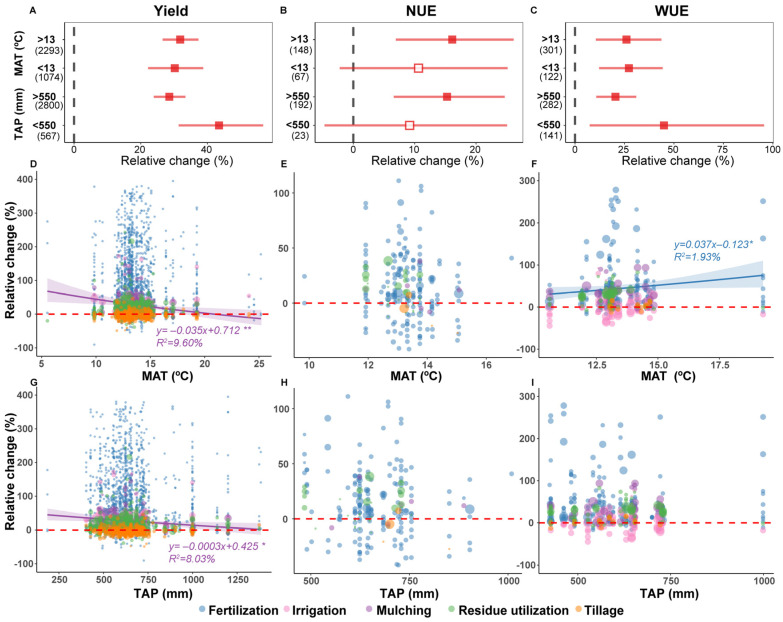
The relative changes in winter wheat yield (**A**,**D**,**G**), NUE (**B**,**E**,**H**), and WUE (**C**,**F**,**I**) response to climate factors under agricultural practices. The numbers in brackets indicated the number of comparisons used for the meta-analysis. Squares with error bars present the means ± 95% confidence intervals, and the solid symbols indicate significant response to agricultural practices; hollow symbols indicate non-significant effects. For regression analysis, the red dashed line represents the line y = 0, the shadow of the corresponding color indicates the 95% confidence interval of the regression line, and the corresponding color labeling is the regression equation and R^2^ value, respectively; * and ** represent *p* < 0.05 and *p* < 0.01, respectively. The size of the circles is proportional to the weight assigned to the study, and the shades of color are caused by the overlap of the circles. TAP, total annual precipitation. MAT, mean annual temperature. NUE, nitrogen use efficiency. WUE, water use efficiency.

Overall, the impact of APs on winter wheat yield showed little variation across different MAT ranges. In relatively arid regions with TAP < 550 mm, APs significantly boosted wheat yield by 43.5% (95% CI: 31.4–56.7%) (Figure 3A). Further regression analysis indicated that the yield increase under mulching gradually decreased with increasing MAT and TAP: for every unit of increase in MAT and TAP, the relative increase in winter wheat yield decreased by 3.5% and 0.03%, respectively (Figure 3D,G). For NUE, APs had no significant effect in cold and arid regions, but in regions with MAT >13 °C and TAP > 550 mm, APs increased winter wheat NUE by 16.1% and 15.3%, respectively (Figure 3B). Conversely, APs enhanced WUE in regions with MAT < 13 °C and TAP < 550 mm, with increases of 27.3% and 45%, respectively (*p* < 0.05, Figure 3C). Under fertilization, the relative change in wheat WUE gradually increased with increasing MAT, with MAT explaining 1.9% of the variance in WUE change (Figure 3F).

### 3.3. Response of Yield, NUE, and WUE to Management Factors Under APs 

**Figure 4:**
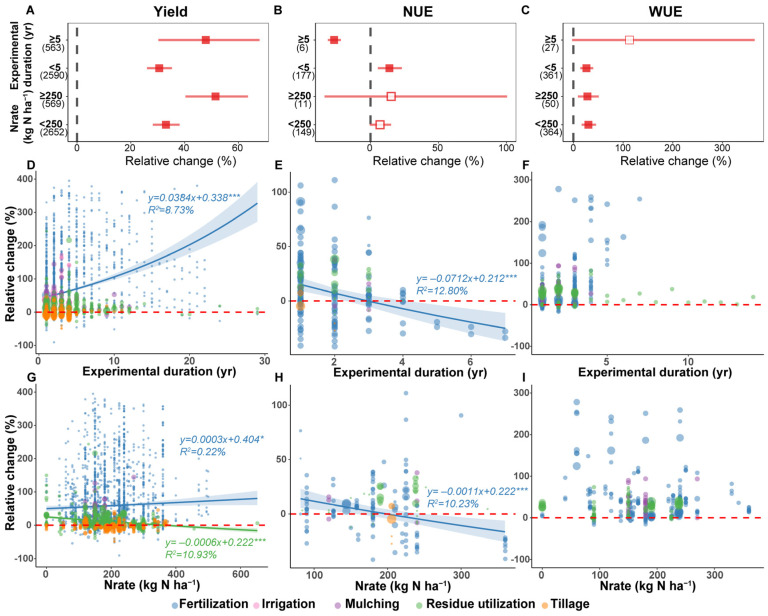
The relative changes in response of winter wheat yield (**A**,**D**,**G**), NUE (**B**,**E**,**H**), and WUE (**C**,**F**,**I**) to management factors under agricultural practices. The numbers in brackets indicate the number of comparisons used for the meta-analysis. Squares with error bars present the means ± 95% confidence intervals, and the solid symbols indicate a significant response to agricultural practices; hollow symbols indicate non-significant effects. For regression analysis, the red dashed line represents the line y = 0, the shadow of the corresponding color indicates the 95% confidence interval of the regression line, and the corresponding color labeling is the regression equation and R^2^ value, respectively; * and *** represent *p* < 0.05 and *p* < 0.001, respectively. The size of the circles is proportional to the weight assigned to the study, and the shades of color are caused by the overlap of the circles. Nrate, nitrogen application rate. NUE, nitrogen use efficiency. WUE, water use efficiency.

The experiment duration significantly impacted the effect of APs on winter wheat yield: when the experiment duration was ≥5 years, the yield increase was highest at 47.9%, while an experiment duration < 5 years yielded an average increase of only 30.6% (Figure 4A). And this time effect was particularly pronounced in regards to fertilization, with the relative increase in yield rising with increasing experiment duration (*p* < 0.001, Figure 4D). In terms of Nrate, the APs with an Nrate ≥250 kg N ha^−1^ showed the best yield increase of 51.5% (95% CI: 40.3–63.5%). Under fertilization, the relative change in wheat yield is positively correlated with Nrate; conversely, the relationship is reversed under residue utilization (Figure 4G). Thus, with 361 kg N ha^−1^ as the threshold, when Nrate < 361 kg N ha^−1^, residue utilization promoted wheat yield increase, while when the Nrate exceeded 361 kg N ha^−1^, residue utilization reduced wheat yield; specifically, the relative change in yield decreased by 0.06% for every 1 kg N ha^−1^ increase in Nrate (*p* < 0.001, Figure 4G, Table S2).

Regarding NUE, the result showed that when the experiment duration was >5 years, APs significantly reduced NUE by 26.7% (*n* = 6), while experiments shorter than 5 years increased NUE by 13.9% (*p* < 0.05, Figure 4B). Among various APs, the relative change in NUE under fertilization was significantly negatively correlated with experiment duration; especially after 3 years, fertilization decreased NUE (*p* < 0.001, Figure 4E). Similarly, fertilization also reduced NUE when the Nrate exceeded 198 kg N ha^−1^, with an approximately 0.11% decreased for every 1 kg N ha^−1^ increase in Nrate (Figure 4H).

For WUE, when the experiment duration was <5 years, AP significantly increased WUE by 26.3% (95% CI: 13.9–40.1%), but its effect became insignificant when the duration was more than 5 years (Figure 4C). And Nrate had little effect on the relative change in WUE under APs; when the Nrate was below and above 250 kg N ha^−1^, WUE increased by 30.3% (95% CI: 16.6–45.6%) and 28.3% (95% CI: 9.1–51.0%), respectively (Figure 4C).

### 3.4. Response of Yield, NUE, and WUE to Soil Factors Under APs 

**Figure 5:**
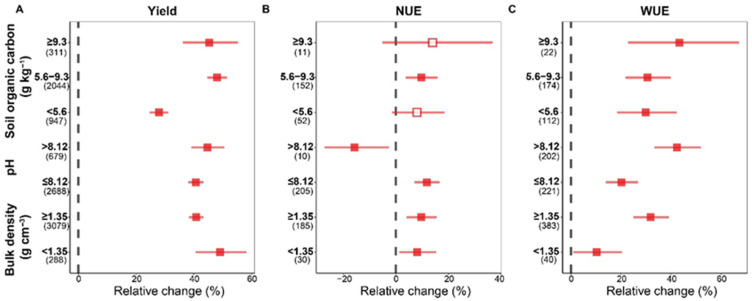
The relative changes in winter wheat yield (**A**), NUE (**B**), and WUE (**C**) response to management factors under agricultural practices. The numbers in brackets indicated the number of comparisons used for the meta-analysis. Squares with error bars present the means ± 95% confidence intervals, and the solid symbols indicated significant response to agricultural practices, hollow symbols indicated not significant effects. SOC, soil organic carbon. BD, bulk density. NUE, nitrogen use efficiency. WUE, water use efficiency.

Soil properties have a crucial impact on the relative changes in winter wheat yield, NUE, and WUE under APs. At different SOC levels, APs significantly increased winter wheat yield; however, in areas with SOC < 5.6 g kg^−1^, the yield increase was relatively smaller (27.9%, Figure 5A). Furthermore, in regions with a SOC of 5.6–9.3 g kg^−1^, APs significantly increased NUE by 9.7% (95% CI: 3.8–15.9%), while in regions with a higher or lower SOC, APs had no significant effect on NUE (Figure 5B). Regarding WUE, as SOC increases, APs enhanced WUE to a greater extent; as shown in Figure 5C, the most pronounced increase was observed when SOC >9.36 g kg^−1^, reaching 43.3% (95% CI: 22.8–67.2%).

The increase in wheat yield under APs showed little variation across different soil pH levels, ranging 40.7–44.8% (Figure 5A). However, the NUE under APs was significantly affected by soil pH: the relative change in NUE was increased by 11.8% when pH < 8.3, while it decreased by 15.7% when pH > 8.3 (Figure 5B). For WUE, APs produced a more pronounced improvement effect in strongly alkaline soils, with an increase of 42.3% (95% CI: 33.4–51.9%) (Figure 5C).

In regions with BD < 1.35 g/cm^3^, APs induced a higher yield increase of 49.2% (95% CI: 40.7–58.3%) (Figure 5A). In contrast, in regions with BD > 1.35 g/cm^3^, AP showed more pronounced improvements in NUE and WUE, particularly for WUE (31.9%, 95% CI: 25.0–39.1%) (Figure 5B,C).

**Figure 6:**
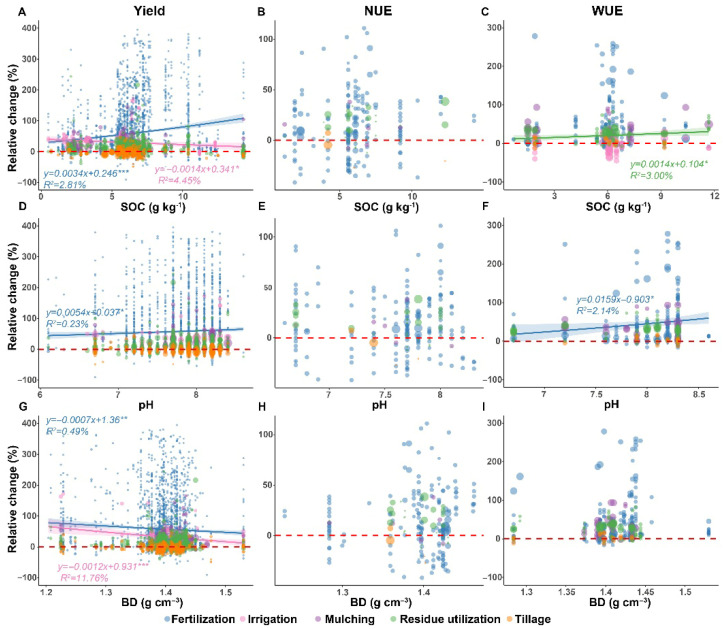
The relationship between relative changes in winter wheat yield (**A**,**D**,**G**), NUE (**B**,**E**,**H**), and WUE (**C**,**F**,**I**) with soil factors under agricultural practices. For regression analysis, the red dashed line represents the line y = 0, the shadow of the corresponding color indicates the 95% confidence interval of the regression line, and the corresponding color labeling is the regression equation and R^2^ value, respectively; *, **, and *** represent *p* < 0.05, *p* < 0.01, and *p* < 0.001, respectively. The size of the circles is proportional to the weight assigned to the study, and the shades of color are caused by the overlap of circles. SOC, soil organic carbon. BD, bulk density. NUE, nitrogen use efficiency. WUE, water use efficiency.

In various APs, the relative change in yield and WUE under fertilization exhibit greater sensitivity to soil factors. Specifically, the yield changes induced by fertilization are positively correlated with SOC and pH, but negatively correlated with soil BD (*p* < 0.01)—for every 1 g cm^−3^ increase in BD, the relative increase in wheat yield decreased by approximately 0.07% (Figure 6A,D,G, Table S2). Similarly, the relative change in WUE under fertilization increased with increasing soil pH (Figure 6F). Furthermore, under irrigation, the increased wheat yield gradually decreased with increasing SOC and BD, for every unit increase in SOC or BD, the increase in wheat yield decreased by approximately 0.14% and 0.12%, respectively (Figure 6A,G, Table S2). For residue utilization, the relative change in winter wheat WUE changes are significantly positively correlated with SOC; for every 1 g kg^−1^ increase in SOC, the relative change in WUE increased by 0.14% (Figure 6C).

### 3.5. Drivers of Relative Changes in Yield, NUE, and WUE Under APs

**Figure 7:**
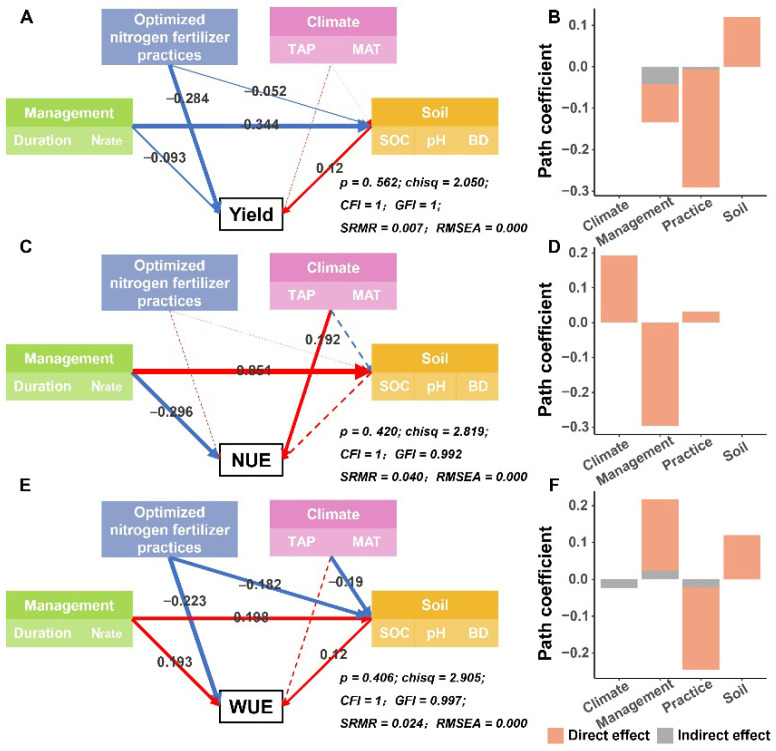
Multi-factors relationships influencing the relative changes in yield (**A**,**B**), NUE (**C**,**D**) and WUE (**E**,**F**) under agricultural practices. Dashed lines indicate insignificant relationships, while solid lines indicate statistical significance (*p* < 0.05); red lines represent positive effects, and blue lines represent negative effects; the numbers marked on the line indicate standardized path coefficient, and the width of the line indicates the size of the path coefficient. BD, soil bulk density. Duration, experiment duration. TAP, total annual precipitation. MAT, mean annual temperature. Nrate, nitrogen application rate. pH, soil acidity. SOC, soil organic carbon content. CFI, comparative fit index. GFI, goodness of fit index. RMSEA, root mean square error of approximation. SRMR, standardized root mean square residual. NUE, nitrogen use efficiency. WUE, water use efficiency.

The largest effect on the relative change in yield was caused by APs, with a standardized effect size (r; higher values of r mean greater effect size) of −0.290; management factors followed (r = −0.134), comprising both direct effects (r = −0.093) and indirect effects (r = −0.041) (Figure 7A,B). Also, management factors were the main driver of the relative change in NUE (r = −0.296; Figure 7C,D). Furthermore, APs had the greatest impact on the relative change in WUE (r = −0.245), followed by management factors (r = 0.217, Figure 7E,F).

## 4. Discussion

This discussion primarily explores three key aspects. First, we explored the impact of APs on wheat yield, NUE, and WUE in a wheat–maize rotation system. Based on this, using regression analysis, we correlated the relative changes in yield, NUE, and WUE caused by APs with variables such as climate, soil, and management, further revealing the mechanisms by which these variables influence the relative changes. Finally, we analyzed the interrelationships among the multiple variables driving these changes. These analyses help clarify the specific role of APs in improving the yield and efficiency of wheat production. Furthermore, while completing the above explorations, this study also systematically reviews the study limitations, aiming to provide clear directions for future research.

### 4.1. The Effect of APs on Wheat Yield

The use of science-based APs is key to enhancing winter wheat yields [34,35], and these are also important drivers of the relative change in yield (Figure 7). This study, through integrated data analysis, clearly revealed the differences in the effect of different APs on winter wheat yield, providing important evidence for further optimizing farmland management strategies. Fertilization maximally promoted winter wheat yield increases, and this conclusion was based on all fertilization-related treatments in current agricultural production (Figure 2A). Compared to no fertilization, COF achieved the largest yield increase. This may be attributed to its synergistic effect of organic and inorganic nutrients, which not only rapidly met crop nutrient demands during critical growth stages but also improved soil physicochemical properties [36], enabling more efficient and sustained nutrient supply [37]. Moreover, in addition to conventional fertilization, biochar addition also demonstrated the strongest yield-promoting effect, consistent with the results of field experiments conducted on winter wheat in the Loess Plateau by Li et al. [38]. As a porous material, biochar’s unique structure effectively improves soil structure and enhances water and fertilizer retention capacity [39]. More importantly, biochar has a strong nutrient adsorption capacity, reducing nutrient leaching and prolonging fertilizer effectiveness, thus creating a synergistic effect with chemical fertilizers and ultimately increasing yield [40,41].

Furthermore, the positive impacts of irrigation and mulching on winter wheat yield are also crucial (Figure 2A). These results were consistent with those in previous reports, You et al. [42] documented that, over a four-year field trial, the grain composition of winter wheat (i.e., number of spikes per hectare, number of grains per spike, and thousand-grain weight) increased linearly or quadratically with increasing annual irrigation. Mulching (such as plastic film mulching) primarily optimizes the microenvironment for winter wheat growth by inhibiting soil moisture evaporation and regulating root zone temperature [43]. The core mechanism of both APs lies in improving WUE, which is particularly significant in semi-arid or seasonally arid winter wheat producing areas. Coupling analysis with climatic factors further indicated that APs are more effective in increasing yield in relatively cold and arid regions (Figure 3A). Notably, the yield increase of winter wheat under mulching showed a significant negative correlation with MAT and TAP (Figure 3D,G), consistent with previous findings on mulching practices [20]. Our study further emphasized that the selection of APs must fully consider regional climatic conditions and that implementing water-conserving practices such as mulching in arid and semi-arid regions plays a crucial role in ensuring winter wheat production and local food security.

At the same time, we observed that yield increases under APs were highest when the experiment duration was ≥5 years (Figure 4A), likely because the benefits of APs are not simply linearly cumulative, but rather achieved by triggering and driving slow but fundamental benign changes in the soil ecosystem [44]. For example, continuous residue utilization or organic fertilizer application accumulates soil organic matter year by year, promoting the formation of stable soil aggregates [45]. Concurrently, soil microbial and animal communities—such as earthworms—recover and thrive, enabling more efficient fixation, transformation, and slow release of applied fertilizers (particularly chemical fertilizers). Consequently, future agricultural research requires stronger support and deployment of long-term field trials, recognizing these as critical for unraveling the complex dynamics of agroecosystems and addressing future food security challenges under climate change.

Furthermore, the impact of APs on winter wheat yield is also modulated by soil properties (Figure 5). Our results showed that low SOC limited the yield-increasing effects of APs, while low BD promoted winter wheat yield (Figure 5A). Low SOC indicated a deficiency in both chemical and biological soil fertility; thus, even with APs such as fertilization and irrigation, crop roots struggle to efficiently utilize these inputs in such soil environments, limiting yield enhancement effects [45]. Regression analysis also indicated that the yield increase of winter wheat under fertilization significantly increased with increasing SOC content (Figure 6A, *p* < 0.001). Conversely, low soil BD typically indicated loose, porous soil with good aeration and water permeability, which facilitated deeper root penetration and expansion in winter wheat, enabling more efficient capture of water and fertilizer resources [46]. These results also highlighted the interrelationships among various elements of soil health.

It is noteworthy that the no-till practice did not significantly increase winter wheat yield in this study (Figure 5A), which may be because the benefits of the no-till method are long-term and systemic. In the short term, while the no-till method helped improve soil structure and increased surface soil organic matter, it may also lead to negative consequences such as lower seed layer temperature and increased soil compaction, thus offsetting its benefits [47,48]. Therefore, in summary, APs to increase winter wheat yield should prioritize scientific and efficient nutrient management (e.g., organic fertilizer), supplemented by synergistic technologies such as biochar incorporation to improve fertilizer efficiency and optimize water management (irrigation and mulching). Simultaneously, the focus should be on its long-term ecological benefits (such as soil and water conservation, carbon sequestration and emission reduction) rather than short-term yield effects, and a comprehensive evaluation should be conducted in conjunction with regional climate and soil conditions during its promotion.

### 4.2. The Effect of APs on Wheat NUE

Improving NUE is one of the most effective means to enhance crop productivity while reducing environmental degradation [49]. Our results indicated that fertilization improved NUE in winter wheat, primarily through SCRF and inhibitor addition, both of which directly positive affect nitrogen transformation processes (Figure 2B). This finding highlighted that, compared to indirectly influencing nitrogen fate by improving the external environment of crop growth, directly regulating the rate and form of nitrogen transformation in the soil at its source, directing more exogenous nitrogen fertilizer to crop absorption, is a more direct and effective method for improving NUE [50]. In contrast, other APs (such as irrigation, residue utilization, or mulching) did not significantly improve NUE (Figure 2B), which may be because while these practices improve soil environment, they fail to directly and precisely intervene in the key pathways of nitrogen transformation; thus, their indirect positive effects on NUE may be offset by the inherent strong nitrification and denitrification processes in the soil.

Our results also indicated that the impact of fertilization on winter wheat NUE is significantly negatively correlated with the experiment duration (Figure 4E). This may be because long-term fertilization without adequate carbon replenishment can disrupt the carbon–nitrogen balance of soil microorganisms, leading to excessive consumption of SOC and degradation of microbial community function [51,52], thereby making it harder for crops to efficiently utilize fertilizers and reducing NUE. Furthermore, regression analysis revealed that the inflection point of winter wheat NUE changes under fertilization: when the Nrate exceeds 198 kg N ha^−1^, the fertilization decreased NUE (Figure 4H), which underscored the importance of rationally controlling nitrogen fertilizer input. Once the nitrogen supply surpasses the maximum demand for crop growth, the crop’s absorption capacity reaches saturation. Excess nitrogen cannot be assimilated and instead accumulates in the soil, leading to losses through leaching as nitrate nitrogen, emissions as nitrous oxide, or volatilization as ammonia [53], ultimately reducing NUE. Therefore, the 198 kg N ha^−1^ may represent a critical threshold at which APs shifts from “pursuing maximum yield” to “pursuing maximum efficiency”.

Furthermore, the positive effect of APs on winter wheat NUE was more significant in regions with higher MAT and TAP (Figure 3B). This may be because warmer and wetter conditions are more conducive to soil microbial activity and nutrient mineralization [54], so APs (such as fertilization and irrigation) can more precisely match water and nitrogen supply with crop demand, reducing growth stress and nutrient loss. Moreover, the impact of APs on NUE also depends on the experiment duration; at the 5-year threshold, APs enhanced NUE in short-term experiments but exhibited negative impacts in long-term experiments (Figure 4B). This may be because some APs may have water and fertilizer retention effects in the short term, but long-term implementation may lead to deteriorated soil physical structure (e.g., increased BD, poor aeration) or imbalanced nutrient cycling [55,56], and these slowly accumulating negative effects outweigh their short-term benefits and become the decisive factor in NUE decline. Notably, the observations for long-term experiments (>5 years) in this study were limited (*n* = 6); therefore, this conclusion requires further verification by more long-term trials. Nevertheless, these findings underscored that the evaluation and adoption of APs must be grounded in long-term, systematic monitoring to prevent short-term gains from misleading long-term decisions, thereby ensuring the synergistic achievement of food security and ecological sustainability goals.

Meanwhile, the effect of APs on NUE is also related to soil physical and chemical properties. In regions with moderate SOC, AP has a significant positive impact on NUE (Figure 5B). This may be because when SOC is too low, the soil’s water and fertilizer retention capacity and microbial activity are low, making it difficult for APs to overcome this fundamental limitation in the short term; conversely, when SOC is too high, the soil itself may already possess a strong nutrient supply and buffering capacity, making it difficult for APs to produce additional significant gains. Regarding soil pH, in strongly alkaline soils with pH > 8.3, APs significantly reduced winter wheat NUE (Figure 5B), which may be attributed to the following: (1) high pH significantly exacerbated ammonia volatilization loss, causing substantial external nitrogen to dissipate into the atmosphere before being absorbed by crops [57]; and (2) sodium ion toxicity accompanying high pH deteriorated the soil structure, severely inhibiting root development and nutrient absorption [58,59]. Finally, in soils with higher BD (>1.35 g cm^−3^), the effect of APs on NUE was relatively more pronounced (Figure 5B). In conclusion, this study emphasized that in intensive agricultural systems pursuing high crop yields and efficiency, compared to the results for continuously increasing fertilizer inputs, regulating fertilizer properties at the source is more effective in improving NUE; simultaneously, establishing synergy between short-term efficient nitrogen utilization and long-term soil health is essential for achieving sustainable agricultural development.

### 4.3. The Effect of APs on Wheat WUE

Our results showed that APs were the main drivers of changes in WUE (Figure 7E,F). Compared to no fertilization, both conventional nitrogen fertilizer and COF substantially increased WUE, consistent with the work of Chen et al. [60]. The underlying mechanism involves fertilization effectively promoting crop growth and biomass accumulation, thereby converting more limited water into economic yield, achieving a physiological synergistic effect of “fertilizer–water regulation” [61]. Additionally, residue utilization and plastic film mulching effectively suppress soil water evaporation, retaining more moisture in the crop root zone [62]. In particular, straw incorporation rapidly increased soil organic matter, promoted aggregate formation, and further enhanced soil water-holding capacity [63].

When assessing the impact of APs on wheat WUE, their interaction with climatic factors must be considered. The effect of APs on WUE enhancement is more pronounced in cold and arid regions (Figure 3C), which may be because such practices effectively alleviate water and temperature constraints, yielding higher marginal benefits and maximizing WUE improvement. Additionally, the relative increase in WUE under fertilization is positively correlated with MAT (Figure 3F). In cold climates, winter wheat photosynthesis and growth metabolism are constrained, preventing efficient conversion of fertilizers and water into biomass [64]; as MAT increases, the abundant nutrients supplied by fertilization are fully utilized by vigorously growing crops, promoting biomass accumulation and directly driving the increase in WUE.

Furthermore, the effect of APs on WUE is significantly influenced by key soil properties. Specifically, APs enhanced WUE more substantially in soils with high SOC (Figure 5C), and the relative increase in WUE under residue utilization gradually increased with increasing SOC (Figure 6C). This may be because high SOC soils have better aggregate structure and pore composition, which effectively intercept and retain water and nutrients [65], so crops can access water and nutrients more sustainably and stably, converting each unit of water resources into biomass more efficiently. Meanwhile, APs also show a more significant WUE-improving effect in alkaline soils and soils with higher BD (Figure 5C). These results collectively highlight the close relationship between the potential of APs to enhance WUE and the soil, providing an important theoretical basis for implementing targeted agricultural practices in different soil types and achieving efficient water resource utilization.

### 4.4. Limitations

This study, based on a constructed comprehensive dataset, systematically evaluated the impact of APs on winter wheat yield, NUE, and WUE in a wheat–maize rotation system using meta-analysis. The strengths of this study are mainly reflected in two aspects: first, we focused on the comprehensive impact of different APs on winter wheat yield, NUE, and WUE, which helps to screen the best APs for increasing efficiency and yield in the context of current global climate change; second, we revealed the variability patterns of APs effects influenced by climate, soil, and management factors and identified the key driving factors, providing scientific basis for government departments and managers to formulate effective, locally adapted agricultural strategies. However, this study retains certain limitations. (1) Geographical limitations of data sources: although we collected as many relevant published observations as possible, the wheat–maize rotation system has a greater planting advantage in China, so the included research mainly comes from within China, which may limit the universality of the conclusions. Therefore, we look forward to more relevant global studies being published in the future to expand our research database and further validate our results. (2) Insufficient data for some APs: although the overall effects of different APs were analyzed, the sample size for some practices is limited (e.g., only six pairs of NUE observations, with experiment durations exceeding 5 years). Meanwhile, only a few studies reported soil types, which hinders our ability to conduct more detailed analyses to explore the advantageous roles of different APs in different soil types.

In summary, this study systematically clarified the pivotal role of APs in enhancing winter wheat production efficiency, along with their regulatory factors, which provided theoretical foundations and practical pathways for implementing synergistic green production models that boost both yield and efficiency in intensive farming regions primarily relying on wheat–maize rotation systems. Future research should further improve the scientific foundation of agroecosystem management and promote sustainable agricultural development by strengthening long-term fixed-site observation, expanding network comparisons across different ecological zones, and deepening research on the coupling mechanism of microbial processes and nutrient cycling.

## 5. Conclusions

This study systematically evaluated the effects of APs on winter wheat yield, NUE, and WUE in a wheat–maize rotation system. Among all APs examined, fertilization synergistically increased winter wheat yield, NUE, and WUE by 43.671%, 16.875%, and 44.698%, respectively, which highlighted the substantial potential for synergistically enhancing crop production and resource efficiency through precision fertilization, holding significant practical value for advancing sustainable food production. And this study further revealed the significant regulatory role of climate, soil, and management factors regarding the effects of APs, meanwhile clarifying that APs were the main drivers influencing yield and WUE changes, while NUE was primarily regulated by management factors. These findings provide key scientific evidence for developing differentiated resource management strategies and have important practical guiding value for promoting sustainable and intensive agriculture under complex environmental conditions.

## Figures and Tables

**Table 1:** Classification of agricultural practices and corresponding treatment and control groups in the meta-analysis.

Agricultural Practices	Treatment	Control	Description of Treatment
Irrigation	Irrigation	No irrigation	Increase soil moisture availability.
	Add irrigation	Conventional irrigation	Increase soil moisture.
Fertilization	Mineral fertilizer	No fertilization	Application of mineral fertilizers, such as N fertilizer,
	Organic fertilizer	No fertilization	including animal manure (such as cow, sheep, chicken) and fermented compost (the mixture of manure and straw, sludge, kitchen waste. etc.).
	Combined organic and mineral fertilizer (COF)	No fertilization	Application of both organic and mineral fertilizers.
	Organic fertilizer	Mineral fertilization	Application of animal manure (such as cow, sheep, chicken), fermented compost (the mixture of manure and straw, sludge, kitchen waste, etc.).
	COF	Mineral fertilization	Application of both organic and mineral fertilizers.
	Biochar	No biochar	Increase the soil carbon pool by converting non-recalcitrant carbon to recalcitrant carbon through pyrolysis of biomass.
Tillage	No tillage	Traditional tillage	No tillage before winter planting to reduce soil disturbance.
Residue utilization	Straw mulching	No residue return	After harvesting of crops, the residues are applied directly on the soil surface.
	Straw incorporation	No residue return	After harvesting of crops, the residues are incorporated into the soil by tillage.
Mulching	Plastic film mulching	No mulching	Plastic film is spread on the soil surface.
	Grass mulching	No mulching	Grass matter is spread evenly on the soil surface.

Data extraction from the studies involved: (1) location (longitude, latitude, and altitude); (2) climatic conditions (MAT, TAP); (3) experiment information (APs, Nrate, experiment duration, and replicates); (4) soil properties: pH, soil BD, SOC content. We extracted data from tables and text directly and utilized GetData Graph Digitizer (https://getdata-graph-digitizer.com/, accessed on 30 September 2025) to extract data presented in figures. For the above data that could not be obtained, we used ArcGIS to extract them from the published datasets based on latitude and longitude, among which the soil data from the SoilGrid (https://soilgrids.org/, accessed on 30 September 2025) and climate data from the World Climate Database (http://www.worldclim.org/, accessed on 30 September 2025) and the site-specific soil data showed very little variance from this dataset, which had little or no effect on the results.

## Data Availability

The raw data supporting the conclusions of this article will be made available by the authors on request.

## Supplementary Materials

The following supporting information can be downloaded at: https://www.mdpi.com/article/10.3390/plants15040617/s1, Text S1: Publication Bias [66]. Text S2: Fit index for structural equation model (SEM); Figure S1: Preferred reporting items for meta-analyses (PRISMA) flow chart describing the article selection process for eligible articles to include in the meta-analysis; Figure S2: Frequency distribution of the response ratios of winter wheat yield (A), NUE (B) and WUE (C). The black dashed lines are fitted normal (Gaussian) distributions to frequency datasets. NUE, nitrogen use efficiency. WUE, water use efficiency; Table S1: Test for publication bias for the response of winter wheat yield, NUE and WUE to agricultural practices using Fail-Safe Analysis with Rosenberg method. Table S2: The results of regression analysis between the relative change in variables with among factors.
