# Supplementary material for: Synergistic Improvement in Wheat Yield, Water and Nitrogen Use Efficiency in Wheat–Maize Rotation Systems: A Meta-Analysis of Multidimensional Agricultural Practices
Source: Plants (Basel). 2026 Feb 15;15(4):617. doi: 10.3390/plants15040617 (PMC12944248; doi:10.3390/plants15040617)
Supplement: Supplementary file 1 [file plants-15-00617-s001.zip › plants-4126637-supplementary.pdf]

Supplementary Information

Synergistic Improvement in Wheat Yield, Water and Nitrogen Use Efficiency in Wheat–Maize Rotation Systems: A Meta-Analysis of Multidimensional Agricultural Practices

Huihui Wei [1,*], Tingting Gong [1], Li Zhou [2] and Li Qin [2,*]

[1] Institute for Interdisciplinary Innovation Research, Xi'an University of Architecture and Technology, Xi'an 710055, China
[2] Institute of Agro-Environmental Protection, Ministry of Agriculture and Rural Affairs, Tianjin 300191, China
* Correspondence: weihh@xauat.edu.cn (H.W.); ql-tj@163.com (L.Q.); Tel./Fax: +86-18434800581 (H.W.); +86-13820976292 (L.Q.)

Supplementary Text

Text S1. Publication Bias

In this study, we used the fail-safe number (Nfs) to detect publication bias in the data. The fail-safe number method was proposed by Rosenthal in 1979 [66]. The core of this method is as follows: when meta-analysis results are statistically significant, what is the minimum number of unpublished studies required to render those meta-analysis results insignificant? The number of unpublished studies here is the Nfs. A larger Nfs means that a large number of unpublished and insignificant studies are needed to render the meta-analysis conclusion insignificant. In other words, a larger Nfs indicates a lower probability of the research conclusion being altered and a less severe publication bias problem. The most commonly used standard is currently "5*n* + 10", meaning that a Nfs greater than 5*n* + 10 indicates that publication bias is not severe, where n is the number of effect sizes.

Text S2. Fit index for structural equation model (SEM)

In this study, the following four indexes were employed to test the fit of the structural equation model (SEM).

(1) CFI (comparative fit index)

$$CFI = 1 - \frac{\max(\chi_t^2 - df_t, 0)}{\max(\chi_b^2 - df_b, \chi_t^2 - df_t, 0)} \quad (1)$$

where χ_t^2 and df_t represent Chi-squared and freedom degrees of the target model, respectively, while χ_b^2 and df_b denote Chi-squared and freedom degrees of the baseline

model (typically an independent model/null model, assuming all variables are independent), respectively. The CFI ranges from 0 to 1, with values closer to 1 indicating better model fit. Typically, CFI ≥ 0.90 is considered acceptable fit, and ≥ 0.95 indicates good fit.

(2) GFI (goodness of fit index)

$$GFI = 1 - \frac{tr\left[\left(S - \hat{S}\right)W^{-1}\right]^2}{tr\left[\left(S\right)W^{-1}\right]^2} \tag{2}$$

where W is the weight matrix (or S when using maximum likelihood estimation). The GFI ranges from 0 to 1, with values closer to 1 being preferable; traditionally, GFI ≥ 0.90 is considered acceptable.

(3) SRMR (standardized root mean square residual)

$$SRMR = \sqrt{\frac{2\sum_{i=1}^{p}\sum_{j=1}^{i}(S_{ij} - \hat{\sigma}_{ij})^2 / (S_i S_j)}{p(p+1)}} \tag{3}$$

where p is the number of variables; Sij and $\hat{\sigma}_{ij}$ are the observed and regenerated covariance matrices, respectively; and Si and Sj are the observed standard deviation. SRMR ranges from 0 to 1, with values closer to 0 being preferable, indicating smaller mean standardized residuals. SRMR ≤ 0.08 is generally considered acceptable, and ≤ 0.05 indicates a good fit.

(4) RMSEA (root mean square error of approximation)

$$RMSEA = \sqrt{\max \frac{\chi^2 - df}{(N-1)df}, 0} \quad (4)$$

where $\chi 2$ and *df* are the Chi-square and freedom degrees of the theoretical model, respectively, and *N* is the sample size. RMSEA measures the difference between the theoretical model and a perfectly fitted saturated model in the population (i.e., approximation error), assessing the average fit difference per freedom degree. Therefore, compared to indexes like GFI, RMSEA is less affected by sample size and remains reliable even with large samples. When RMSEA is ≤ 0.05, it represents a good fit, meaning that the SEM differs little from the data; when $0.05 < \text{RMSEA} \leq 0.08$, it represents a reasonable fit, meaning that the SEM has acceptable approximation error; when $0.08 < \text{RMSEA} \leq 0.10$, it represents a normal fit, and the SEM may need improvement; when $\text{RMSEA} > 0.10$, it represents a poor fit, meaning the model has serious problems.

Supplementary Figure

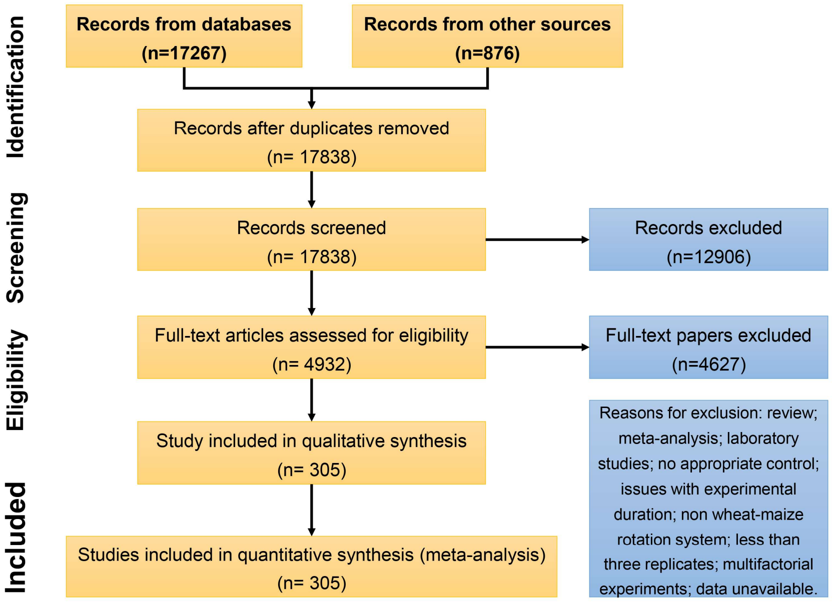

Figure S1. Preferred Reporting Items for Meta-Analyses (PRISMA) flow chart describing the article selection process for eligible articles to be included in the meta-analysis.

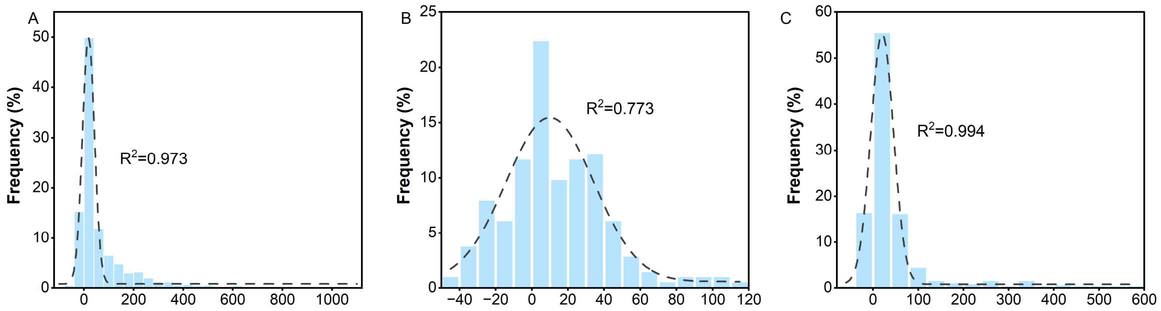

Figure S2 Frequency distribution of the response ratios of winter wheat yield (A), NUE (B), and WUE (C). The black dashed lines show normal fitted (Gaussian) distributions to frequency datasets. NUE, nitrogen use efficiency. WUE, water use efficiency.

Supplementary Table

Table S1. Test for publication bias for the response of winter wheat yield, NUE, and WUE to agricultural practices using fail-safe analysis with the Rosenberg method.

Variable	*n*	Fail-Safe Coefficient
Yield	3368	>5*n*+10
NUE	215	>5*n*+10
WUE	423	>5*n*+10

Note: *n* is sample size. For fail-safe analysis, coefficients > 5*n*+10 indicated that the effect sizes of the variables are statistically significant, and there was no potential publication bias in the dataset. NUE, nitrogen use efficiency. WUE, water use efficiency.

Table S2. The results of regression analysis between the relative change in variables and among factors.

Variable	Factor	Agricultural Practice	Intersection Point with y=0
Yield	MAT (°C)	Mulching	19.5
Yield	TAP (mm)	Mulching	1350
Yield	Nrate (kg N ha^{-1})	Residue utilization	361
NUE	Experiment duration (yr)	Fertilization	3
NUE	Nrate (kg N ha^{-1})	Fertilization	198

Text: NUE, nitrogen use efficiency. WUE, water use efficiency. Nrate, nitrogen fertilizer application rate. TAP, total annual precipitation. MAT, mean annual temperature. Nrate,

nitrogen application rate.

Reference

66. Rosenthal, R., The file drawer problem and tolerance for null results. Psychol Bull 1979, 86, (3), 638.
